# Bio-Corrosion of Magnesium Alloys for Orthopaedic Applications

**DOI:** 10.3390/jfb8030038

**Published:** 2017-09-01

**Authors:** Emily K. Brooks, Mark T. Ehrensberger

**Affiliations:** 1 Department of Biomedical Engineering, University at Buffalo, Buffalo, NY 14214, USA; ekbrooks@buffalo.edu; 2 Department of Orthopaedics, University at Buffalo, Buffalo, NY 14214, USA

**Keywords:** magnesium, alloy, corrosion, orthopaedics, biomaterial

## Abstract

Three Mg alloys, Mg–1.34% Ca–3% Zn (MCZ), Mg–1.34% Ca–3% Zn–0.2% Sr (MCZS), and Mg–2% Sr (MS), were examined to understand their bio-corrosion behavior. Electrochemical impedance spectroscopy and polarization scans were performed after 6 days of immersion in cell culture medium, and ion release and changes in media pH were tracked over a 28 day time period. Scanning electron microscopy (SEM) of alloy microstructure was performed to help interpret the results of the electrochemical testing. Results indicate that corrosion resistance of the alloys is as follows: MCZ > MCZS > MS.

## 1. Introduction

Metallic biomaterials commonly employed in orthopaedic applications include titanium and its alloys, stainless steels, and a class of cobalt–chromium–molybdenum based alloys. Although these devices offer superior mechanical strength, they are bioinert, and permanently remain as a foreign material in the body. It is not uncommon that a second surgery for removal of the device, especially in the case of fracture fixation, is necessitated [1]. In order to improve upon the current treatment standards, magnesium (Mg) and its alloys are being investigated for their use as biodegradable implant materials. A magnesium implant would be intended to fully degrade, eliminating the need for a second surgery for device removal. Mg degrades through a series of corrosion reactions when exposed to the physiological environment, producing byproducts, including Mg^2+^ ions, OH^−^, and H_2_. Previously published work has provided a detailed review of the suggested Mg corrosion mechanisms [2,3].

Magnesium possesses mechanical properties similar to those of bone tissue [4]. As the fourth most abundant mineral in the body, Mg is an essential element, and over half of its total content is stored in bone [5]. The presence of Mg^2+^ ions has been shown to increase cell adhesion and osteoblastic activity in vitro [6,7,8,9], and in vivo material implantation results in improved bone regeneration and healing [10,11,12]. Recent studies have reported potential antimicrobial properties associated with Mg degradation [13,14,15,16].

Although these advantages make Mg and its alloys promising candidates for use as orthopaedic materials, their widespread clinical use has been hindered, based on low corrosion resistance in the physiological environment. Mg corrosion is accelerated in chloride containing solutions [17], and a high concentration of chloride ions in the body combined with a neutral buffered pH, limits the formation of Mg(OH)_2_ as a partially protective byproduct, leaving Mg materials susceptible to continued corrosion. Uncontrolled Mg corrosion can have detrimental effects in vivo, where it is possible for released hydrogen gas to accumulate in the surrounding tissue, as well as OH^−^ ions increasing the local pH [18]. Efforts made to control the corrosion of Mg in a biological environment include alloying and applying protective surface coatings [19]. In terms of alloying, much of the initial focus was given to Mg–aluminum (Al) alloys, including AZ91 [20] and AZ31, due to their commercial availability. However, it has since been recognized that Mg–Al alloys should be avoided in biological applications, as aluminum release in the body may be related to the development of dementia or Alzheimer’s [18]. 

More recently, a range of Mg alloys have been created and tested specifically for their use as biomaterials in both orthopaedic and cardiovascular applications [21]. Some progress has been made in alloying Mg with rare earth elements (REs) to slow material corrosion in a physiological environment [11]. But it must considered that these elements do not naturally exist in the body, and that their long term effects are still unknown [22]. Short term in vitro work has shown that some REs may be more suitable than others, and that careful consideration to composition must be given [23]. 

In order to avoid concerns associated with potential toxicity, Mg alloying elements should be restricted to those that have already demonstrated long-term biocompatibility. For this reason, three Mg alloys will be focused on herein: Mg–1.34% calcium–3% zinc (MCZ), Mg–2% strontium (MS), and Mg–1.34% calcium–3% zinc–0.2% strontium (MCZS). Zinc (Zn) may lead to increased bone formation through enhancing alkaline phosphatase and collagen production [24], and calcium (Ca) ions have been correlated to enhanced osteoblast proliferation and differentiation in vivo [25]. Strontium (Sr) is also recognized as an osteogenic factor, and may induce the differentiation of mesenchymal stem cells towards the osteoblastic lineage [26,27]. Ideally, incorporation of Ca, Zn, and Sr in a material may further enhance the bone-forming response to a Mg alloy implant. Beyond enhancing biological properties, alloying elements may also contribute to a mechanically stronger material. Both Mg alloyed with Sr and Zn [28], as well as Mg alloyed with Ca and Zn [29], have displayed improved mechanical performance compared to pure Mg. However, alloying additions to improve osteogenic properties and mechanical strength often increase the corrosion rate of the Mg material. 

The goal of the study was to characterize the bio-corrosion of three Mg alloys containing combinations of Ca, Zn, and Sr in vitro, to contribute to the development of Mg materials for biomedical applications. Previous studies have shown MCZ to display acceptable in vitro corrosion rates [30], and this alloy will act as a control in our investigation. On the other hand, conflicting results have been presented concerning the investigation of Mg–Sr alloy systems. Gu et al. found that Mg_2_Sr performed the most favorably when testing a range of Sr additions [31], whereas others have suggested limiting Sr additions to 0.5%, in order to avoid rapid corrosion [32]. MS is studied in attempts to clarify the impact of higher Sr loading on Mg alloy corrosion. Finally, studies on a novel alloy, MCZS, will help to elucidate the effects of a fourth element addition on the corrosion of MCZ. Corrosion processes of the materials were characterized over a 28 day in vitro immersion period in a physiologically relevant medium.

## 2. Results

### 2.1. Microstructure

**Figure 1:**
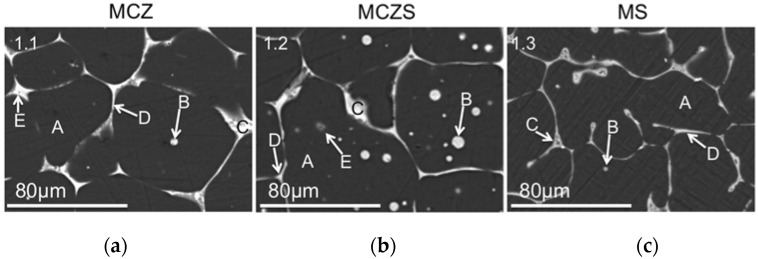
Backscattered SEM images of the MCZ (**a**), MCZS (**b**), and MS (**c**) alloys after polishing. Elemental mapping of the imaged area was completed (see Figure 2). Regions marked A, B, C, D, and E represent the areas where composition was analyzed using EDS (energy dispersive X-ray spectroscopy). Results of the EDS analysis are presented in Table 1.

Figure 1 displays the scanning electron microscopy (SEM) images of the alloys obtained after polishing to a mirror finish. The grain structure varies considerably between the alloys; MCZ displays the most continuous grain boundaries, while MS presents a less defined grain structure. MCZS exhibits an intermediate structure with grains seemingly larger than MCZ, but not as well defined. Intergranular particles are apparent in both MCZ and MCZS, with their size and frequency increasing in the MCZS alloy.

**Figure 2:**
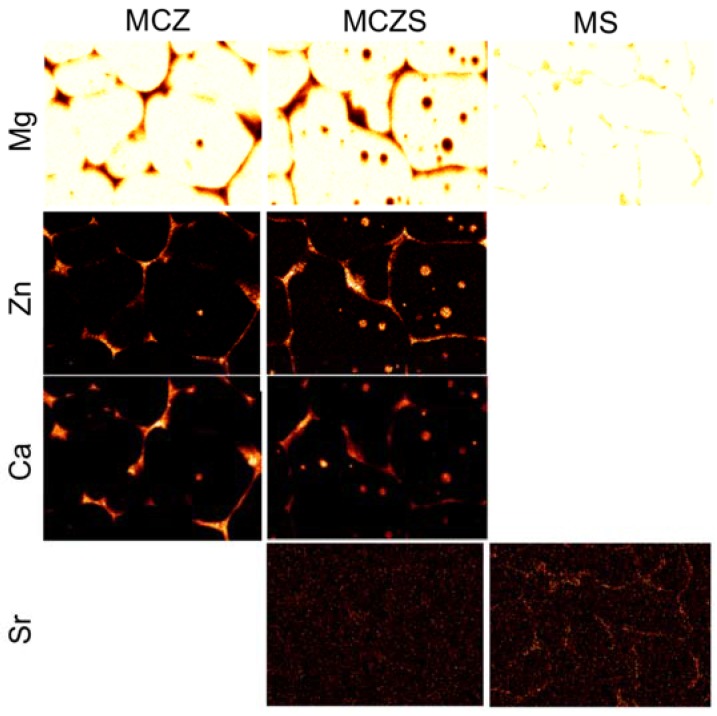
Distribution of alloying elements in MCZ, MCZS, and MS as determined by elemental mapping. Original backscatter SEM images of the areas that were mapped for each alloy are displayed in Figure 1.

EDS point analysis corresponding to the regions indicated in Figure 1 is presented in Table 1. The matrix of the MCZ alloy is dominated by Mg, with a small amount of Zn also dispersed throughout the α-Mg matrix and limited presence of Ca (Figure 1a, area A). The intergranular particles are composed similar to the matrix, but contain slightly higher concentrations of Ca (Figure 1a, area B), while the second phase formed at the intersection of the grain boundaries contain considerably higher concentrations of Zn and Ca (Figure 1a, areas E, C). The MCZS alloy has a similar bulk Mg matrix, with no Sr present (Figure 1b, area A). The intergranular particles and grain boundaries again contain higher concentrations of the alloying elements (Figure 1b, areas B, C, D, E). A second type of intergranular particle could be detected in MCZS alloys (Figure 1b, area E), which contained a higher concentration of Ca than Zn or Sr. MS again showed no presence of Sr in the α-Mg matrix (Figure 1c, area A), but rather, the Sr was found concentrated in a second phase along the grain boundaries (Figure 1c, area C, D). The distribution of elements in the alloys was further confirmed with elemental mapping (Figure 2), which emphasized the high concentration of alloying elements present in the grain boundaries and intergranular particles.

### 2.2. Solution Analysis

#### 2.2.1. pH Measurements

**Figure 3:**
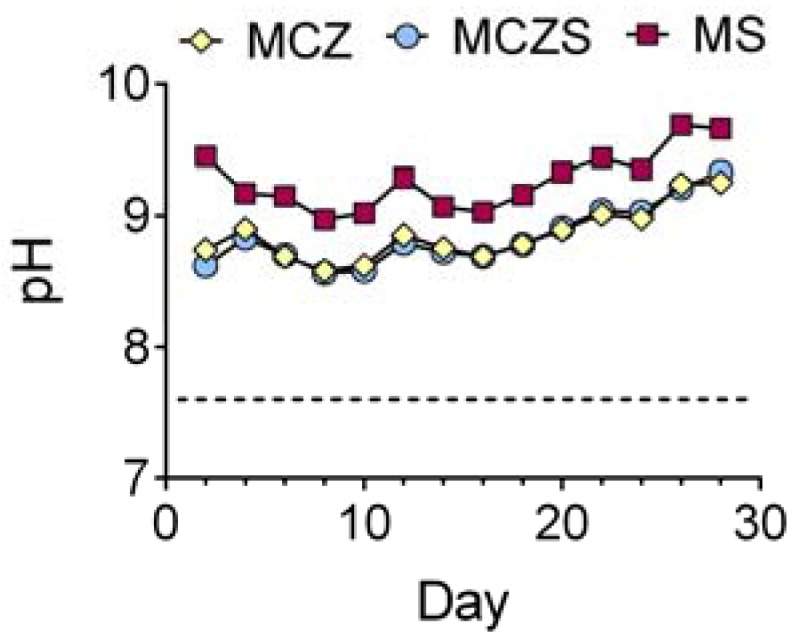
Trends in pH of the test electrolyte measured at each media exchange every 48 h over the 28 day immersion period. Results are plotted as mean ±1 standard deviation. The dotted line represents the pH of the fresh CCM (cell culture medium) substituted into the chambers during the media exchanges.

Although the immersion media contained a sodium bicarbonate buffer system, Mg alloy corrosion was still able to produce an alkaline shift in the solution (Figure 3). Media was replenished every 48 h, at which point in time the pH of the spent media was measured and recorded. MCZ and MCZS showed similar trends in pH, slowly increasing to reach levels just above 9 over the 28 day immersion period. MS consistently displayed a greater alkaline pH than the other alloys.

#### 2.2.2. ICP-MS

**Figure 4:**
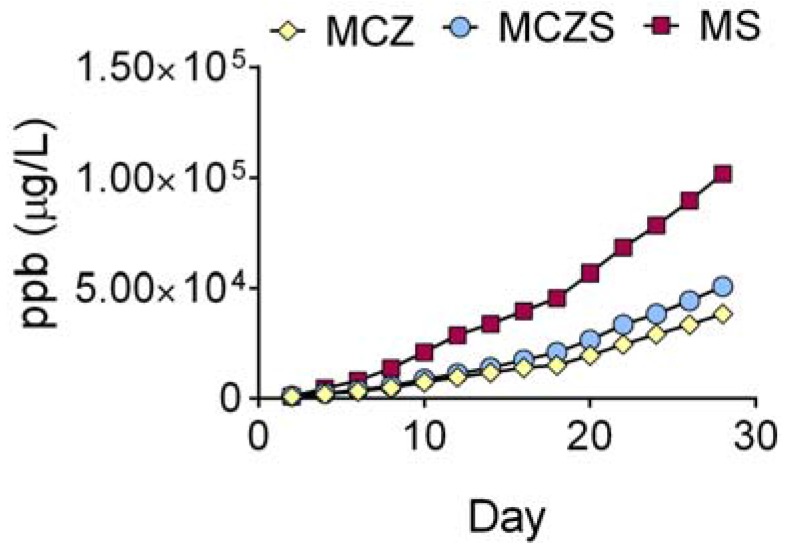
Cumulative ion release over the 28 day immersion period as measured by ICP-MS. Plotted data represent the sum of released elements each media exchange.

Cumulative ion release as measured by inductively coupled plasma mass spectroscopy (ICP-MS) corresponds to the trends observed for the pH measurements. The results (Figure 4) reflect the summation of Mg ions and alloying elements for each material. The total ion release increases gradually and continuously for all alloys, with MS showing greater concentrations of ions released to solution when compared with MCZ and MCZS (Figure 4). 

**Figure 5:**
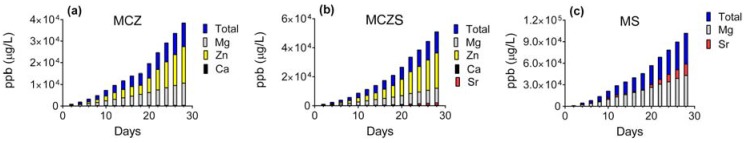
Cumulative ion release over the 28 day immersion period as measured by ICP-MS. The data display the release of individual elements for MCZ (**a**), MCZS (**b**), and MS (**c**).

Figure 5 shows the ICP-MS results for each material separately, displaying the specific ion release in μg/L measured for the applicable constituent elements of the alloys. It can be seen that both Mg and alloying elements are being released from the materials over time. The increasing concentration of alloying elements, along with Mg present in the electrolyte, emphasizes the possible local degradation of the second phase and intermetallics. 

### 2.3. Electrochemical Measurements

#### 2.3.1. Open Circuit Potential

The open circuit potential (OCP) of the alloys shows a shift towards increasingly electropositive potentials over time (Table 2). The initial OCP measured is more electronegative for MS compared to MCZ and MCZS; however, after 6 days of immersion, the OCP of all materials has settled to around −1.5 V.

#### 2.3.2. Polarization Scans

**Figure 6:**
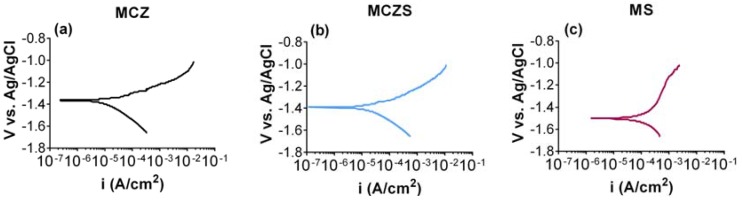
Representative polarization scans of MCZ (**a**), MCZS (**b**), and MS (**c**) after 6 days of immersion in the cell culture medium.

Representative polarization scans of each alloy after 6 days of immersion in cell culture medium are presented in Figure 6. The E_corr_ and I_corr_ derived from the polarization scans are presented in Table 3. It is understood that in general, a smaller I_corr_ and more electropositive E_corr_ indicate enhanced corrosion resistance of a material. Both MCZ and MCZS have a significantly smaller I_corr_ and are more electropositive E_corr_ when compared to MS. These results point to the MS alloy being the most susceptible to corrosion.

#### 2.3.3. EIS

**Figure 7:**
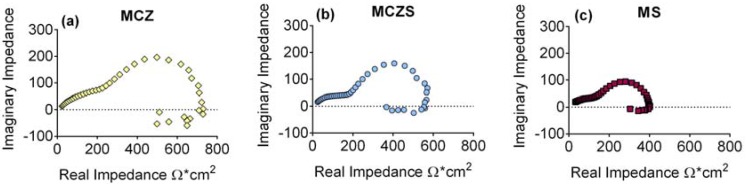
Representative Nyquist plots of MCZ (**a**), MCZS (**b**), and MS (**c**) after 6 days of immersion in the cell culture medium.

**Figure 8:**
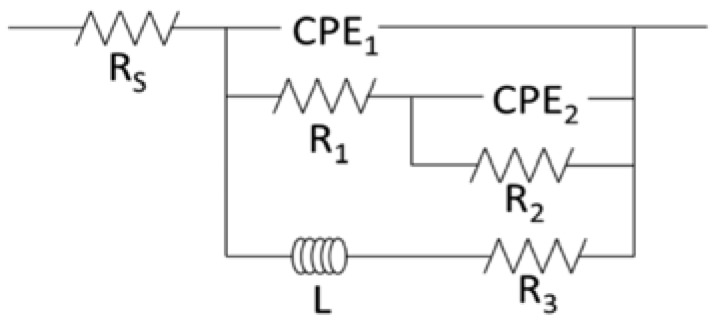
The equivalent circuit model used to fit EIS data from the three alloys generated after 6 days of immersion. Abbreviations are as follows: R_S_, solution resistance; CPE_1_, double layer capacitance; R_1_, resistance related to initial metallic corrosion; CPE_2_, pseudo-capacitance; R_2_, resistance to discharge an intermediate; L, an inductor; R_3_, resistance related to the inductor.

Nyquist plots corresponding to the EIS (electrochemical impedance spectroscopy) spectra collected for the Mg alloys after 6 days of immersion in cell culture medium are displayed in Figure 7. The plots show similar shapes regardless of material; all contain two capacitive loops at high and medium frequencies, and an inductive loop at low frequencies. However, the diameter of these capacitive and inductive loops varies between the alloys, indicating differences in the material’s resistance to corrosion. The EIS results were fit to a nested two time constant circuit model including an inductor (Figure 8). The circuit chosen to fit the data has previously been justified as an accurate model for corrosion of Mg [33], and been show to apply to its alloys [34]. Constant phase elements (CPE) are included to better represent deviations from ideal capacitive behavior caused by an inhomogeneous surface. A CPE is defined by both its magnitude, Q, an indication of the capacitive behavior, and its exponent α, a representation of the surface heterogeneity.

Within the circuit model, R_S_ corresponds to the electrolyte resistance, CPE_1_ models the capacitance of the double layer, and R_1_ is related to the initial metallic corrosion. The remaining circuit elements can be assigned a physical meaning based on Mg corrosion taking place through a mechanism involving intermediate surface coverage dependent on potential [34,35]. CPE_2_ is related to an adsorption pseudo-capacitance whose value is determined by the charge stored in the intermediate film [33,36,37], while R_2_ models the resistance to discharge of the intermediate [36]. The final branch of the circuit, containing the inductor (L) and R_3_, represents the presence of a second adsorbed intermediate able to enhance material corrosion [33]. Results of the fitting procedure (Table 4) then allow for calculation of the polarization resistance (R_P_) using Equation (1) [33]:

(1) $$R_{P}=\frac{1}{\frac{1}{R_{1}+R_{2}}+\frac{1}{R_{3}}}$$

*R_P_* is inversely related to the corrosion current, and is an important parameter in understanding the electrochemical behavior of a material. 

The EIS results show that MCZ has greater resistance to the initial charge transfer step (R_1_) than MS, and greater resistance to discharging of the intermediate (R_2_) than both MS and MCZS. Q_2_ is significantly greater for both MCZ and MCZS in comparison to MS, perhaps indicating the adsorbed intermediate has an increased capacity for charge storage on these materials. Finally, the calculations for *R_P_* suggest that out of the investigated alloys, MCZ displays significantly greater resistance to corrosion processes than MS, with MCZS at intermediate levels.

### 2.4. Comparison of Corrosion Rates

Table 5 shows the corrosion rates calculated for the Mg alloys after 6 days of immersion in the test electrolyte. For polarization scan samples, the corrosion rate was determined from the Tafel analysis. The Stern Geary relationship was applied to the calculated *R_P_* from EIS, and Faraday’s Law was used to convert the ion release measured from ICP-MS to a corrosion current. 

The results do display variability in calculated corrosion rate based on the method used. Our ICP-MS results are an average corrosion rate over the initial 6 days of immersion, while the Tafel and EIS results reflect the instantaneous corrosion rate when the test was carried out on day 6 of immersion. Further ICP-MS results may be affected by the likelihood that ions can remain attached to the surface as surface films, and may not all be released to the electrolyte. Therefore, this technique may underestimate the charge corrosion rate of the Mg alloys. Although we have taken precautions to fully analyze the EIS results at the low frequency region, it appears *R_P_* may have been overestimated, again resulting in lower than expected measured corrosion rates. The importance of properly modeling EIS data to obtain a reasonable *R_P_* has been well explained in the literature [33]. 

### 2.5. Surface Morphology

**Figure 9:**
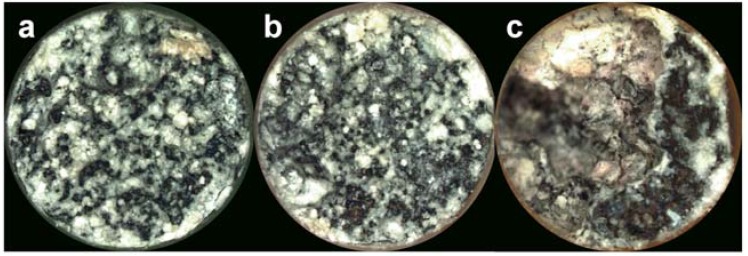
The surface of MCZ (**a**), MCZS (**b**), and MS (**c**) after 28 days of immersion in cell culture medium. The field width of view for the images is 6 mm.

Figure 9 displays representative stereoscope images of the surface morphology of the Mg alloys at the conclusion of 28 days of immersion in CCM. The entire surface of each of the alloys exhibits evidence of corrosion as well as corrosion product formation likely indicated by the white precipitates on the surface. The corroded surface and product formation appear similar for both MCZ and MCZS, while alloy MS shows evidence of severe corrosion. A portion of the alloy appears to have completely degraded, resulting in a deep opening into the sample.

## 3. Discussion

Alloying elements chosen for Mg alloys can have significant effects on both their corrosion behavior and biocompatibility [38]. It is critical to characterize and fully understand the degradation processes Mg alloys will display in the physiological environment before their clinical use becomes widespread. Results from the presented EIS and polarization testing are in agreement, and indicate that the corrosion resistance is as follows: MCZ > MCSZ > MS. The calculated *R_P_* for MCZ is statistically increased compared to MS, and the I_corr_ for both MCZ and MCZS is lower compared to MS. In general, the quaternary alloy containing all elements, MCZS, displays intermediate corrosion behavior. Although the corrosion rates calculated for the various methods employed here are not quantitatively in close agreement with each other, they emphasize similar trends in the data.

Additionally, both electrolyte measurements and examination of surface morphology confirm the results of electrochemical testing. MS corrosion produces a more substantial alkaline shift in pH when compared to MCZ and MCZS. The OH^−^ ions responsible for the increase in pH are generated as a byproduct of the water reduction reaction, the cathodic reaction involved in corrosion of Mg. Therefore, increased electrochemical reaction rates will generate a higher concentration of OH^−^ ions, ultimately leading to a greater alkaline shift in pH. Along with this, we observed greater total ion release for MS compared with MCZ and MCZS, again indicating increased material dissolution, likely through enhanced anodic activity. Finally, visual examination of the alloys following the 28 day immersion period confirmed that all materials experienced some degree of corrosion, with MS showing macroscopic signs of severe degradation. 

Microstructure is known to be a key determinant in the corrosion behavior of Mg and its alloys [39,40], and is likely a factor in the high corrosion rates measured for MCZ, MCZS, and MS. One common result of alloying Mg is grain refinement, which has been suggested to affect Mg corrosion in different ways. In one case, it is possible that grain refinement can support decreased corrosion rates through creation of a surface film with fewer defects [39]. A more refined and complete grain structure may also be able to reduce corrosion by acting as a physical barrier to the progression of corrosion across the surface of the material [41]. However, it is also recognized that an increase in the volume of the second phase, often located along the grain boundaries, can severely lower the corrosion resistance of Mg alloys [40]. We believe that the alloy loading in our materials increased the volume of the second phase resulting in excessive corrosion.

Second phases formed in Mg alloys are generally electropositive, and more noble, compared to the Mg matrix. This allows the second phase to support cathodic reduction reactions, while simultaneously enhancing microgalvanic corrosion between the bulk matrix and additional phases of the alloy [40]. Microgalvanic corrosion is likely an important factor for each of the alloys tested, as we observed the presence of additional phases containing concentrated alloying elements in each material. 

For MS, the second phase, Mg_17_Sr_2_, is known to form along the grain boundaries of magnesium–strontium alloys, increasing as the concentration of strontium increases [31]. A Mg–2% Sr alloy has shown lower corrosion rates compared to pure Mg, Mg–1% Sr, Mg–3% Sr, and Mg–4% Sr, as well as acceptable in vivo performance [31]. But, others have suggested lower Sr alloying amounts (~0.5%) are necessary to avoid rapid material degradation [28,42]. SEM imaging confirmed the presence of localized regions with high Sr content in the MS alloy studied. Accelerated corrosion in areas immediately next to the Mg_17_Sr_2_ phase has previously been documented [43], and the increased measured corrosion rate for MS compared with MCZ and MCZS may be due to the presence of second phase Mg_17_Sr_2_ supporting high rates of galvanic corrosion. Further, our initial measurements of OCP show a more electronegative value for MS compared to the other alloys, which is expected for Sr addition to a Mg alloy [40], and indicated a more active material. It is possible, that, as suggested by Brar et al. [28], lower concentrations of Sr may produce an alloy with more favorable corrosion resistance. 

The tertiary and quaternary alloys investigated contain equal additions of Zn and Ca, with Sr addition to the MCZS alloy limited to 0.2%, in an attempt to avoid the rapid material degradation suggested to occur with higher alloying concentrations of Sr [32]. Magnesium–zinc–calcium alloys were focused on as they contain elements known to be biocompatible, and have shown promise in terms of tailoring corrosion rates of Mg materials for biomedical applications. This possibility stems from the ability for Zn additions to control the cathodic reaction rate of the material, while Ca additions control the anodic reaction rate [29]. It must be noted that the Ca additions were kept at the solid solubility limit of 1.34%, in an attempt to avoid rapid dissolution generated by Mg_2_Ca phase formation. Calcium is an active metal, and unlike other alloying elements in that any Mg_2_Ca formed will maintain a negative potential in comparison to the Mg matrix, resulting in substantial increases in corrosion [40,44]. 

MCZS was investigated as a novel quaternary alloy. MCZS showed significantly decreased corrosion rates when compared to MS as determined by a lower I_corr_, and was further found to demonstrate only slight increases in corrosion when compared to MCZ. No statistical differences were detected in the I_corr_ or *R_P_* of the two materials, and the measured OCP was comparable at all time points. However, the ion release of MCZS was slightly higher than that of MCZ. This may once again result from differences in microstructure of the two materials, as the addition of 0.2% Sr increased both the size and frequency of intermetallic particles found in MCZS compared to MCZ. The ratio of cathodic area to anodic area is known to play an important role in galvanic corrosion. An increasing volume of the more electropositive second phase would increase the area ratio of the cathode in comparison to the bulk matrix anode for MCZS versus MCZ, resulting in increased anodic current, needed to maintain charge conservation in the system. Although both alloy MCZ and MCZS showed improved performance compared to MS, fast corrosion rates were still likely initiated by microgalvanic corrosion between phases of the material. 

The presented results emphasize the importance of continued development of Mg alloys for biomedical applications. The results of the current study serve as evidence of increased corrosion rates caused by high alloy loading in Mg materials. Moving forward, investigations should focus on materials with decreased alloying additions, to avoid rapid corrosion rates observed in the work presented herein. 

## 4. Materials and Methods

### 4.1. Sample Preparation

Mg alloys were custom produced using a vacuum induction furnace, where pure Mg was processed, along with the master alloys of elements of focus. The crucible, stirrer, mold, and thermocouple protector, were coated with a graphite spray to minimize Fe contamination. The crucible was first held at 200 °C for 5 min to remove any moisture. The furnace chamber was evacuated to approximately 0.01 bar, and backfilled with argon to create an inert atmosphere. The alloy was heated at approximately 3–5 °C/s to 720 °C, and then held for 25–30 min, while the molten alloy was stirred multiple times. The molten alloy was then poured into a mold preheated to 200 °C to minimize shrinkage. Subsequent to casting, the alloys were processed by homogenization and quenching, followed by extrusion and rolling. Mg alloys were then machined into 6 mm diameter rods, from which samples were cut for testing, and individually embedded in epoxy resin. The samples were sequentially wet sanded to a 600-grit finish, sonicated in 70% ethanol for 10 min, and placed under ultraviolet (UV) light for 30 min, for sterilization.

### 4.2. Scanning Electron Microscopy (SEM)

SEM was used to examine the microstructure of the alloys. Samples were further prepared by incrementally polishing with glycol diamond suspensions to 1 μm, followed by final polishing with 0.04 μm colloidal silica suspension. Samples were sputter coated with carbon and examined in backscattered electron (BSE) mode with a SEM (Hitachi SU70, Tokyo, Japan) equipped with energy dispersive X-ray spectroscopy (EDS) using mapping technique and point analysis. 

### 4.3. Evaluation of Corrosion Behavior

#### 4.3.1. Test Electrolyte

In order to simulate the conditions of the human body, a cell culture medium (CCM) containing proteins and a bicarbonate buffer was used as the test electrolyte for corrosion studies. The CCM was composed of alpha minimum essential medium (αMEM, Gibco, Grand Island, NY, USA) supplemented with 10% fetal bovine serum (fetal bovine serum (FBS), Atlanta Biologicals, Flowery Branch, GA, USA), and 1% penicillin/streptomycin (Gibco) [45]. A sodium bicarbonate (NaHCO_3_) buffer system was present in the αMEM, which should maintain the pH of the solution at physiologically relevant levels, near 7.4. The test electrolyte was replenished every 48 h over the duration of all immersions in an attempt to maintain relevant near neutral pH levels. 

#### 4.3.2. Corrosion Chamber Preparation

A custom three-electrode corrosion cell was used to assess the electrochemical behavior of the alloys [46]. Resin embedded Mg alloy samples, with an exposed area of 2.8 cm^2^, served as the working electrode, and were positioned between a nylon base and glass tubing with an O-ring providing a watertight seal. 8 mL of the test electrolyte was added to the glass tubing before covering with either a rubber stopper or breathable membrane. The rubber stopper, which included holes for gas exchange, suspension of a graphite counter electrode, and suspension of a silver/silver chloride (Ag/AgCl) reference electrode was used for samples dedicated to electrochemical testing. All potentials reported are with respect to the Ag/AgCl reference electrode. The breathable membrane was used to seal samples for solution measurements. The final surface area to volume ratio for all samples was close to 30 mL/cm^2^, and the corrosion cells were maintained in a humidified cell culture incubator at 37 °C with 5% CO_2_. The test electrolyte was exchanged every 48 h during testing.

#### 4.3.3. Electrochemical Tests

Appropriate connections were made to a potentiostat (Ref 600, Gamry, Warminster, PA, USA) and the open circuit potential (OCP) of the alloys was monitored continuously, over the initial 24 h of exposure, to test the electrolyte. Following 6 days of incubation, electrodes were again connected to a potentiostat. At this point, the OCP was monitored for 20 min, before performing either a polarization scan or electrochemical impedance spectroscopy (EIS). Potentiodynamic polarization scans were executed at a scan rate of 1 mV/s from −0.15 V vs. OCP to 0.5 V vs. OCP. The resulting curves were analyzed to estimate the E_corr_, I_corr_, and corrosion rate (mm/y). EIS was carried out by applying a 10 mV sinusoidal oscillation about the OCP, over a frequency range of 100 kHz to 5 mHz. The resulting impedance data was fit to an appropriate equivalent circuit model using a complex nonlinear least-squares method (EIS 300 software, Gamry Instruments). After modeling the EIS data, *R_P_* was calculated as described in the results section [33]. The Stern–Geary relationship was used to determine a corrosion current, based on the calculated *R_P_* and Tafel slopes derived from the polarization scans [17]. The corrosion current was subsequently converted into a corrosion rate (mm/y) [17]. 

Both polarization scans and EIS were conducted on a minimum of 4 samples of each alloy. The electrochemical tests were considered terminal and incubation of the samples was discontinued following test execution.

#### 4.3.4. Solution Analysis

Three samples of each Mg alloy were maintained in the test electrolyte for 28 days, for analysis of solution pH and ion release. At each exchange of the CCM test electrolyte (performed every 48 h), 1 mL was set aside for pH measurement using a micro-pH meter, and 2 mL aliquots underwent an acid digestion in preparation for analysis by inductively coupled plasma mass spectroscopy (ICP-MS) as previously described [47]. Samples of CCM not exposed to Mg alloys were digested in order to determine the baseline concentration of alloying elements present in the media, which were later subtracted from the experimental results. ICP-MS was performed with a Perkin Elmer Sciex model ELAN DRC-II after calibration with optima grade nitric acid. All ICP-MS samples were analyzed in triplicate.

Measured ion release of the materials after 6 days of immersion (days 0–6) was used to calculate the corrosion current by applying Faraday’s Law (Equation (2)).

(2) $$I_{corr}=\frac{mnF}{tM},$$

where *m* is the released mass in grams, *n* = 2 is the charge transfer number, *F* is the Faraday constant, *t* is the total time in seconds, and *M* is the molecular weight. The calculated current was subsequently converted to a corrosion rate (mm/y) to allow for comparison to results obtained from electrochemical testing.

#### 4.3.5. Surface Morphology

Following the 28 day immersion period, alloy samples used to obtain solution measurements were removed from their corrosion chambers, rinsed with distilled water, and allowed to air dry. The resulting surface morphology of metal samples was then examined with a stereo microscope (Nikon SMZ-U, Tokyo, Japan). 

### 4.4. Statistical Analysis

A Welch’s ANOVA with Games Howell post hoc tests, where appropriate, was used to identify differences in equivalent circuit model elements as determined by EIS, as well as E_corr_ and I_corr_ as determined by the polarization scan. A *p*-value of less than 0.05 was considered significant. Statistical software (SPSS) was used to perform all statistical calculations.

## 5. Conclusions

1. 2% Sr addition to Mg in a binary alloy, MS, is able to cause rapid dissolution of the material, likely through microgalvanic corrosion initiated by increased volume of the second phase Mg_17_Sr_2_.
2. MCZ showed the lowest corrosion rate of the investigated materials.
3. MCZS alloy displayed slightly increased corrosion in comparison to MCZ, but significantly lower corrosion in comparison to MS.
4. All materials investigated showed evidence of fast corrosion in the physiological environment, likely due to microgalvanic corrosion between second phases and intermetallics formed by the high alloy loading.

In order to identify an optimal Mg material for biomedical applications, alloying additions should be reduced during future Mg alloy development.

## Figures and Tables

**Table 1:** Composition (wt. %) of regions of the Mg alloys determined by EDS point analysis. Regions of analysis are indicated in Figure 1.

	MCZ			MCZS				MS	
	Mg	Zn	Ca	Mg	Zn	Ca	Sr	Mg	Sr
Region A	97.93	1.78	0.30	97.89	1.82	0.29	-	100.00	-
Region B	97.85	1.70	0.46	62.38	22.19	13.38	2.05	98.56	1.44
Region C	57.91	26.34	15.75	66.01	22.23	8.52	2.24	82.49	17.51
Region D	98.02	1.61	0.37	88.18	7.26	4.20	0.36	80.47	19.53
Region E	80.74	7.65	11.62	89.76	2.38	7.85	0.01	-	-

**Table 2:** Open circuit potential (OCP) (mean ±1 standard deviation) recorded for each of the Mg alloys at the start of immersion, and after 24 h, and 6 days, of immersion.

Time	MCZ	MCZS	MS
Initial (20 min)	−1.58 ± 0.03	−1.59 ± 0.02	−1.65 ± 0.03
24 h	−1.52 ± 0.00	−1.52 ± 0.00	−1.53 ± 0.03
6 Days	−1.50 ± 0.00	−1.50 ± 0.00	−1.51 ± 0.00

**Table 3:** Corrosion current density (I_corr_) and corrosion potential (E_corr_) (mean ±1 standard deviation) of Mg alloys after 6 days of immersion in cell culture medium determined from the polarization scans.

	MCZ (a)	MCZS (b)	MS (c)	*p*-Value (a vs. b)	*p*-Value (a vs. c)	*p*-Value (b vs. c)
E_corr_ (V vs. Ag/AgCl)	−1.36 ± 0.02	−1.38 ± 0.02	−1.50 ± 0.01	0.430	0.002	0.002
I_corr_ (A/cm^2^)	8.16 × 10^−6^ ± 2.48 × 10^−7^	1.33 × 10^−5^ ± 5.57 × 10^−6^	4.39 × 10^−4^ ± 1.04 × 10^−4^	0.299	0.007	0.008

**Table 4:** Results (mean ± 1 standard deviation) of the EIS circuit model parameters and calculated *R_P_*.

	MCZ (a)	MCZS (b)	MS (c)	*p*-Value (a vs. b)	*p*-Value (a vs. c)	*p*-Value (b vs. c)
R_S_ (Ω·cm^2^)	2.91 ± 2.21	3.48 ± 2.49	5.20 ± 3.09	0.923	0.411	0.613
R_1_ (Ω·cm^2^)	301.68 ± 23.55	253.50 ± 73.94	157.04 ± 19.77	0.416	0.000	0.088
R_2_ (Ω·cm^2^)	473.92 ± 39.06	383.46 ± 13.93	296.80 ± 64.75	0.010	0.004	0.082
R_3_ (Ω·cm^2^)	1860.00 ± 209.76	1676.00 ± 463.44	2218.00 ± 439.74	0.713	0.303	0.201
L (H·cm^2^)	1.41 × 10^4^ ± 5.62 × 10^3^	1.37 × 10^4^ ± 4.25 × 10^3^	2.05 × 10^4^ ± 5.39 × 10^3^	0.993	0.218	0.134
Q_1_ (S·s^a^/cm^2^)	8.17 × 10^−5^ ± 2.85 × 10^−5^	7.06 × 10^−5^ ± 3.07 × 10^−5^	5.32 × 10^−5^ ± 2.07 × 10^−5^	0.828	0.233	0.571
α_1_	0.46 ± 0.02	0.47 ± 0.03	0.47 ± 0.03	0.768	0.848	0.995
Q_2_ (S·s^a^/cm^2^)	1.36 × 10^−4^ ± 2.81 × 10^−5^	1.94 × 10^−4^ ± 3.68 × 10^−5^	7.14 × 10^−5^ ± 5.83 × 10^−6^	0.061	0.013	0.003
α_2_	0.82 ± 0.05	0.85 ± 0.05	0.79 ± 0.01	0.713	0.363	0.116
*R_P_* (Ω·cm^2^)	546.62 ± 28.18	459.99 ± 80.84	376.26 ± 64.04	0.153	0.005	0.228

**Table 5:** Comparison of corrosion rates (mm/y) measured with different methods.

Method	MCZ	MCZS	MS
Tafel Analysis	0.187	0.303	10.024
EIS	0.001	0.001	0.011
ICP-MS	0.011	0.013	0.041
